# Damage progression in rheumatoid arthritis: the role of biologic agents

**DOI:** 10.1186/ar3716

**Published:** 2012-03-08

**Authors:** Ennio Giulio Favalli

**Affiliations:** 1 Chair and Division of Rheumatology, Gaetano Pini Institute, University of Milan, Italy

## 

Given the strict link between structural damage and disability, the prevention of disease progression can be considered the ultimate goal of every rheumatoid arthritis (RA) treatment strategy. At least 4 imaging tools for measuring damage progression in RA are now available: x-rays, ultrasounds, MRI, and CT scan. However, so far in main randomized clinical trials (RCTs) radiography was used as the gold standard for structural damage assessment. Several different radiographic scoring methods have been proposed, but nowadays the most widely used are the Sharp method and its subsequent modifications proposed by van der Heijde and Genant. A recently published paper has demonstrated a strong correlation between various scoring systems. Sixteen main registrative RCTs evaluating the performance of biologic agents in slowing RA structural damage can be found in the literature. Overall, all biotherapies demonstrated a significantly lower damage progression than methotrexate (MTX) used both in monotherapy (adalimumab in the PREMIER and etanercept in the TEMPO trials) and in combination with MTX, with the exception of golimumab in the GO-FORWARD trial. So far, no RCTs head-to-head comparing various biologic agents have been yet performed, and indirect comparisons may have several important limitations regarding study design, study population selection, and choice of used radiographic scoring method. Standardization of scores generated with different scoring systems, matching clinical trials by disease duration and previous treatments, and evaluating the annual estimated progression in each trial may be crucial key factors in order to better compare data coming from different RCTs. Applying this comparison approach, the impact of various biologic drugs in slowing x-ray progression seems to be similar in early RA. On the contrary, the differences in study population characteristics are too marked in late RA for an adequate comparison of biologics performance in this clinical condition. Anyway, the overall impact of biotherapies versus MTX on damage progression seems to be similar in early and in late RA patients. The results in the open extension phase of some previously mentioned RTCs seem to confirm the positive effect of biologic drugs on structural damage in long-term evaluation. A few data coming from RCTs (TEMPO trial) suggest the possibility of repairing bone erosions in patients treated with TNF blockers. Preliminary data on denosumab suggest a possible role of this RANK-L inhibitor in the treatment of RA, mainly as a part of a combination therapy in association with synthetic DMARDs.

